# Short-term acclimation in adults does not predict offspring acclimation potential to hypoxia

**DOI:** 10.1038/s41598-018-21490-y

**Published:** 2018-02-16

**Authors:** Manuela Truebano, Oliver Tills, Michael Collins, Charlotte Clarke, Emma Shipsides, Charlotte Wheatley, John I. Spicer

**Affiliations:** Marine Biology and Ecology Research Centre, Plymouth University, Plymouth, PL4 8AA UK

## Abstract

The prevalence of hypoxic areas in coastal waters is predicted to increase and lead to reduced biodiversity. While the adult stages of many estuarine invertebrates can cope with short periods of hypoxia, it remains unclear whether that ability is present if animals are bred and reared under chronic hypoxia. We firstly investigated the effect of moderate, short-term environmental hypoxia (40% air saturation for one week) on metabolic performance in adults of an estuarine amphipod, and the fitness consequences of prolonged exposure. We then reared the offspring of hypoxia-exposed parents under hypoxia, and assessed their oxyregulatory ability under declining oxygen tensions as juveniles and adults. Adults from the parental generation were able to acclimate their metabolism to hypoxia after one week, employing mechanisms typically associated with prolonged exposure. Their progeny, however, did not develop the adult pattern of respiratory regulation when reared under chronic hypoxia, but instead exhibited a poorer oxyregulatory ability than their parents. We conclude that species apparently hypoxia-tolerant when tested in short-term experiments, could be physiologically compromised as adults if they develop under hypoxia. Consequently, we propose that the increased prevalence of hypoxia in coastal regions will have marked effects in some species currently considered hypoxia tolerant.

## Introduction

Hypoxia is one of the most rapidly changing environmental factors in coastal ecosystems, where oxygen tensions (PO_2_) have decreased dramatically over the past decades as a result of increased anthropogenic nutrient input^1^ and climate change^2^. It is also now among the most widespread deleterious anthropogenic influences, particularly in estuarine and marine environments^3^, which are often subject to seasonal or episodic hypoxia^4^.

Physiologically, the ability to cope with hypoxia depends on the timescale of exposure. In coastal regions, hypoxia tends to be periodic, and there is evidence that physiological responses differ between cyclical and chronic exposure, as well as with severity and time-scales^5,6^. In the short term, hypoxia induces alterations in either metabolism (hypometabolism and/or recourse to anaerobic metabolism), or O_2_ uptake and transport (hyperventilation and/or increased perfusion), which ensure O_2_ delivery to tissues under conditions of low environmental PO_2_ (reviewed in^6,7^). After prolonged exposure, these physiological mechanisms are replaced by more medium- to long-term modifications, including increases in gill surface area, increased concentration and/or affinity of O_2_-carrying molecules, or a combination of these^6,7^. As the extent and duration of hypoxic events are predicted to intensify^8^, it is unclear whether aquatic organisms equipped with mechanisms to cope physiologically with acute and short-term hypoxia will also be able to cope with prolonged or chronic exposure to even moderately low PO_2_.

The increase in extent and duration of hypoxic events in coastal regions means that aquatic invertebrates with rapid generation times will inevitably be exposed to at least moderate hypoxia across their life cycle, yet little attention has been paid to the effect of rearing animals in low PO_2_ (although see^9^). The ability to maintain O_2_ supply upon chronic exposure to low PO_2_ during the whole life cycle, will determine the ability of aquatic fauna to survive, grow and reproduce successfully. Understanding the physiological mechanisms underpinning this ability of aquatic organisms to cope with, acclimate and adapt to reduced PO_2_ is essential if we are to make predictions about the biological and ecological impacts of increasing hypoxia in coastal regions.

We investigated the physiological mechanisms associated with the ability of an aquatic invertebrate to cope with moderate hypoxia in the short-term, and to test for effects of chronic exposure to moderate hypoxia across its life cycle. First, we investigated the effects of short-term (one week) exposure of adults to hypoxia on metabolic performance, as well as the mechanisms underpinning their response. Second, we compared the oxyregulatory ability of these adults, with that of juveniles and adults bred and reared under hypoxic conditions, whose parents were also kept under those conditions at the time of mating. Third, we investigated the fitness consequences of exposure to moderate hypoxia.

Gammarid amphipods are good models as they are abundant in coastal and estuarine areas where they are major components of the food web^10^. The short-term hypoxic response has been documented for a number of gammarid species^11–16^ and varies with environmental history, with species inhabiting highly variable habitats exhibiting the greatest tolerance and best physiological performance^13,17^. Consequently, the brackishwater *Gammarus chevreuxi*^18^ was chosen for study, as it inhabits estuaries, where it experiences marked fluctuations in salinity, temperature and PO_2_.

## Results

### Mechanisms underpinning the ability to maintain metabolic performance in adults exposed to short-term hypoxia

**Figure 1:**
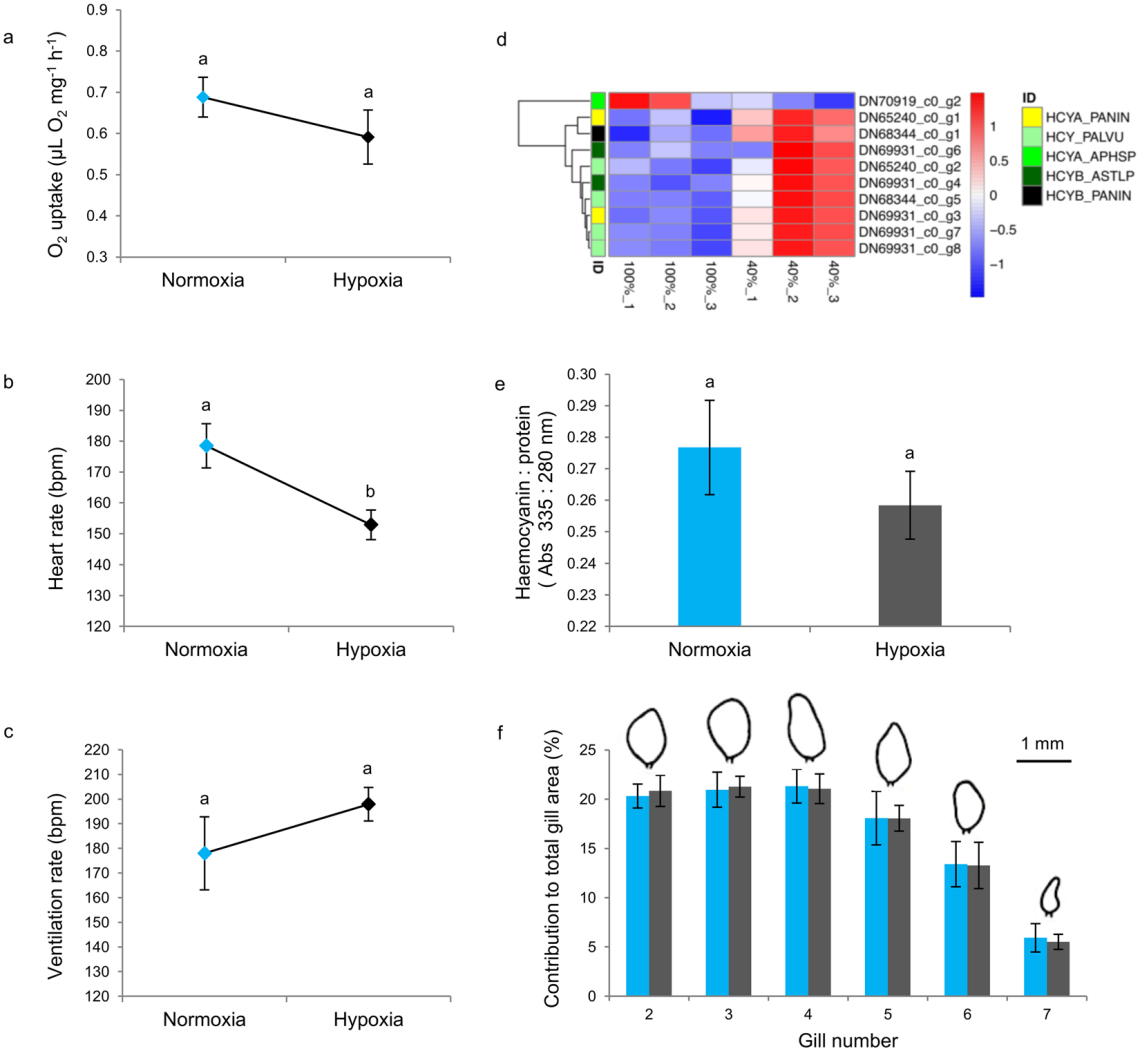
Physiological performance of *Gammarus chevreuxi* under normoxia and moderate hypoxia. (**a**) Rates of mass-specific O_2_ uptake, (**b**) heart rate, (**c**) ventilation rate, (**d**) expression levels (variance stabilised counts) of ten transcripts putatively identified as haemocyanin. ID represents the most significant annotation, (**e**) haemocyanin (λ = 335 nm) to protein (λ = 280 nm) ratios and (**f**) contribution to the total gill area of gills from each pereon segment. Gill form outlines from a representative adult male are given above the bars. Numbers identify pereopod of origin. Amphipods were exposed to either normoxic (100% air saturation) or hypoxic (40% air saturation) conditions for one week. Mechanisms investigated are those typically associated with responses to short-term (hours to days), (**b**–**c**) and prolonged (weeks to months), (**d**–**f**) exposure to hypoxia in crustaceans. Values are expressed as mean ± s.e.m. Letters above bars indicate significant differences between groups (*P* < 0.05).

Exposure to moderate hypoxia (40% air saturation or approx. 2.6 mL O_2_ L^−1^) for one week did not significantly affect the mass-specific rate of oxygen uptake in *G. chevreuxi* (Fig. 1a). The maintenance of oxygen uptake was accompanied by a significant decrease in heart rate (Fig. 1b, t-test, *t*_12_ = −2.96, *P* = 0.012) but no significant changes in ventilation (Fig. 1c). Exposure to moderate hypoxia for one week significantly affected the expression of ten genes putatively identified as haemocyanin, with one gene significantly downregulated under hypoxia, while the remaining nine were significantly upregulated (Fig. 1d, Table S1). There were no significant differences in haemocyanin to protein ratios (Fig. 1e), total gill area, total lamellar area contribution to total gill area, individual gill contribution to total gill area (Fig. 1f) between individuals exposed to normoxia and those exposed to moderate hypoxia for one week.

### Effects of chronic exposure to hypoxia across the life cycle

**Figure 2:**
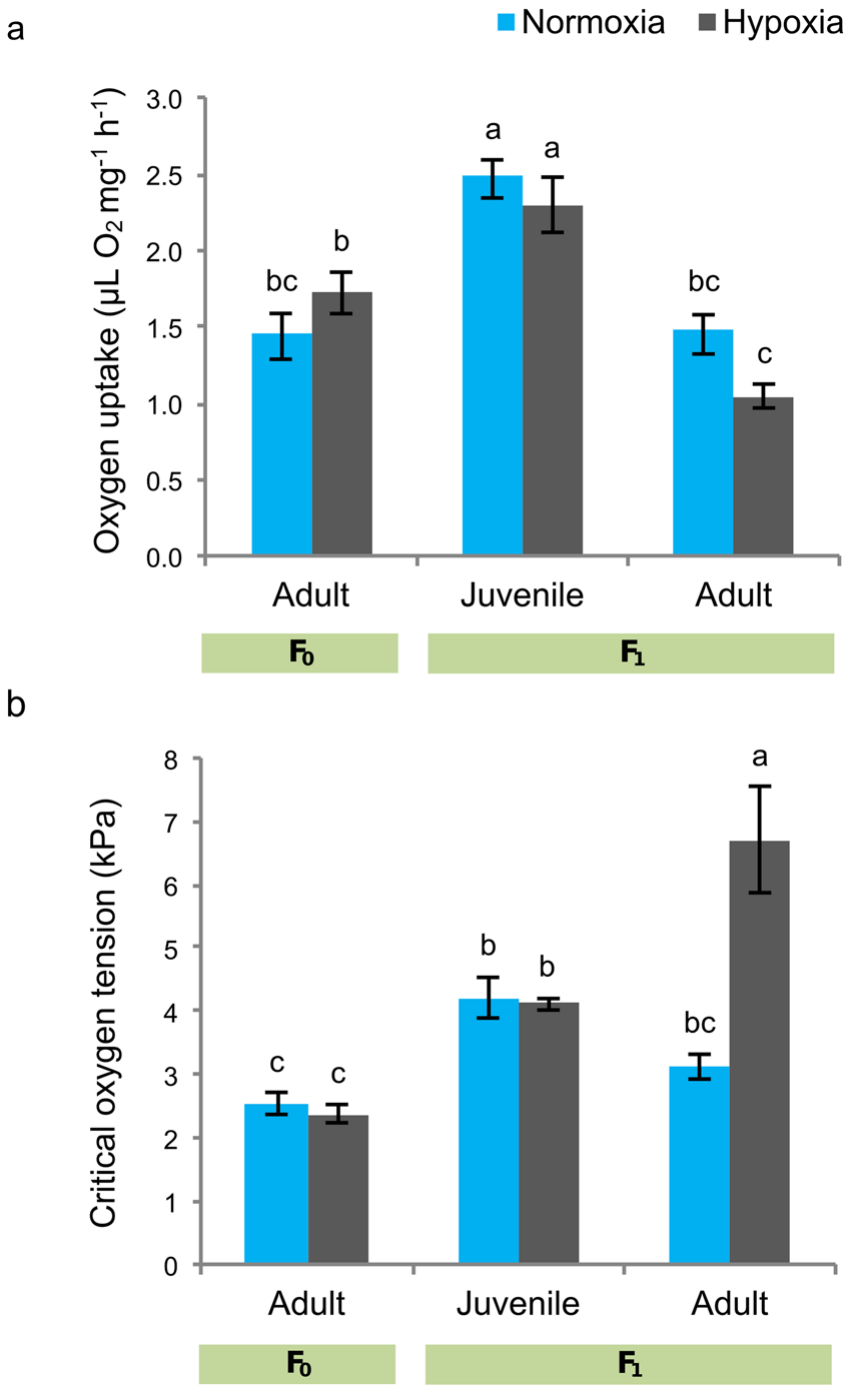
Chronic responses of juveniles and adult *Gammarus chevreuxi* to moderate hypoxia. (**a**) Mass-specific rates of O_2_ uptake under acutely declining PO_2_ and (**b**) critical PO_2_ (or P_c_) of F_0_ adults (n = 6), F_1_ juveniles (n = 5) and F_1_ adults exposed to normoxic (100% air saturation) and hypoxic (40% air saturation) conditions. Individuals from the F_1_ generation were bred from parents exposed to the same treatment as their offspring, and reared under the same conditions. Values are expressed as mean ± s.e.m. Letters above bars indicate significant differences between groups (*P* < 0.05).

Exposure to hypoxia for one week did not affect rates of mass-specific O_2_ uptake or critical PO_2_ (P_c_ or the point at which an individual loses respiratory independence when exposed to acutely declining PO_2_) of F_0_ amphipods. Juvenile F_1_ amphipods bred from hypoxia-exposed parents and reared under hypoxic conditions showed greater rates of O_2_ uptake (Fig. 2a, ANOVA, F_2,27_ = 41.67, *P* < 0.001) and P_c_ (Fig. 2b, ANOVA, *F*_2,27_ = 23.58, *P* < 0.001) than their parents, irrespective of the experimental treatment. When these F_1_ juveniles became sexually mature, i.e. adults, rates of O_2_ uptake and P_c_ were not significantly different from those of their parents in individuals reared under normoxia. However, in F_1_ adults reared under hypoxia from hypoxia-exposed parents, rates of O_2_ uptake were significantly lower than those of their parents (Fig. 2a, ANOVA, life cycle stage, *F*_1,27_ = 41.67, *P* < 0.001, O_2_ regime x life cycle stage *F*_1,27_ = 4.26, *P* = 0.026) and P_c_ significantly higher (Fig. 2b, ANOVA, life cycle stage, *F*_1,27_ = 23.58, *P* < 0.001, O_2_ regime x life cycle stage *F*_1,27_ = 15.77, *P* < 0.001) (Fig. 2, Table S2).

### Measures of fitness-related traits under different O_2_ regimes

**Figure 3:**
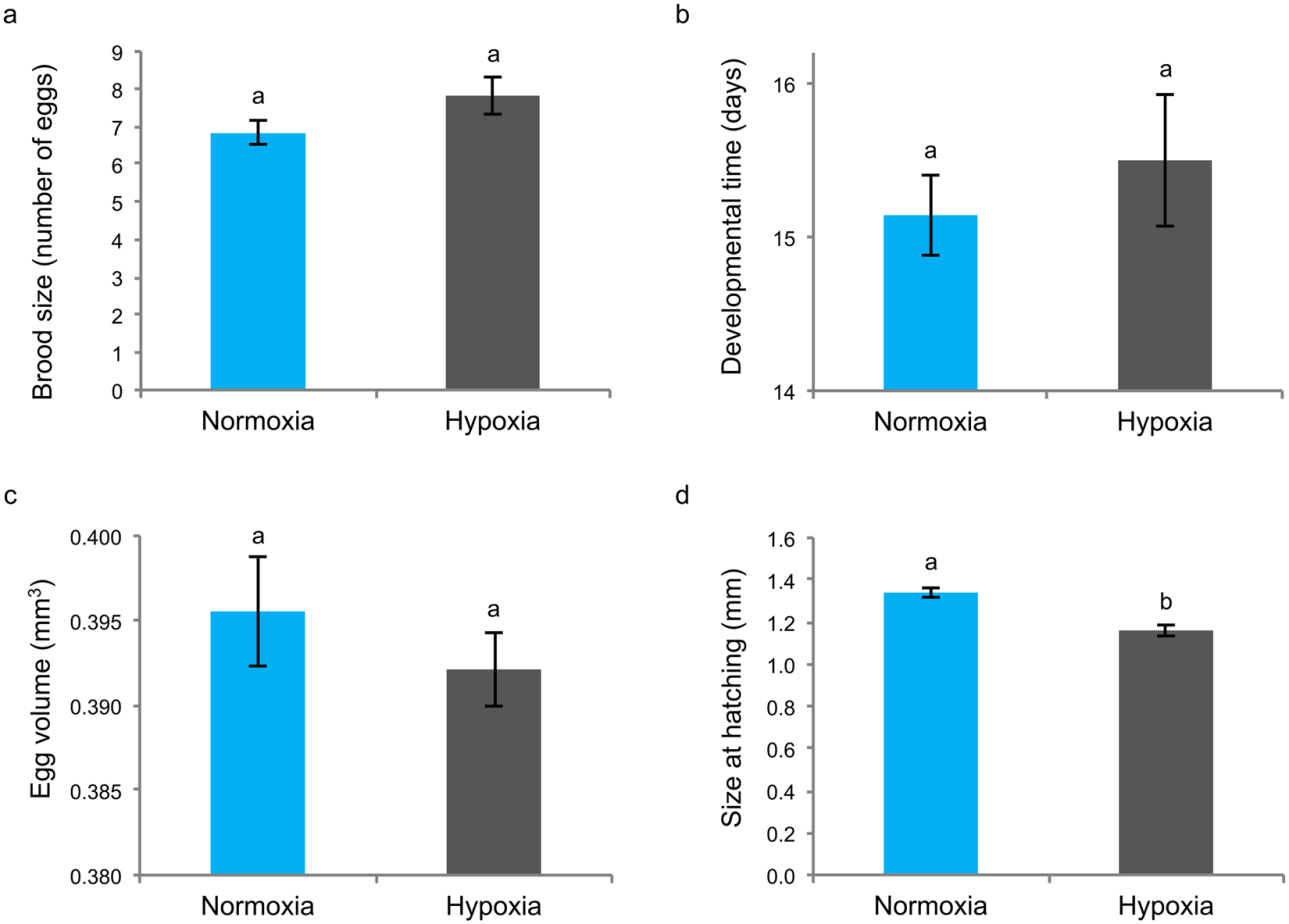
Effect of moderate hypoxic exposure (40% air saturation) on measures of fitness in *Gammarus chevreuxi*. (**a**) Brood size, (**b**) developmental time, (**c**) egg volume and (**d**) size at hatching in F_1_ individuals under normoxia (100% air saturation) and hypoxia (40% air saturation). Values are expressed as mean ± s.e.m. Letters above bars indicate significant differences between groups (*P* < 0.05).

Hypoxic exposure of the F_0_ generation did not significantly affect brood size (Fig. 3a), developmental time (Fig. 3b) or egg volume (Fig. 3c), but did lead to reduced size at hatching in their offspring (Fig. 3d, t-test, *t*_11_ = 5.25, *P* < 0.001). Sexually mature, F_1_ adults reared under hypoxic conditions were significantly smaller than those reared under normoxia (t-test, *t*_14_ = 3.32, *P* = 0.005).

## Discussion

Our results show that exposure to moderate hypoxia can have markedly different effects on metabolic performance depending on whether adults are exposed to short-term hypoxia, or undergo the whole of their development under hypoxic conditions. As the duration and extent of hypoxic areas is predicted to increase in coastal regions, impairment of metabolic performance in individuals reared under moderate hypoxia may affect the resilience of some estuarine species currently considered hypoxia-tolerant.

The brackishwater amphipod, *Gammarus chevreuxi*, maintained normoxic levels of aerobic metabolism when exposed to moderate hypoxia for one week, despite a marked bradycardia (i.e. reduced cardiac activity) and without recourse to hyperventilation (i.e. increased pleopod rate). While the bradycardia could be maladaptive, it could also be interpreted as part of an energy-saving mechanism and/or be associated with an increase in stroke volume in order to maintain or even increase cardiac output, as in some decapod crustaceans^19,20^. A significant negative correlation between heart rate and aerobic metabolism under hypoxia (*R*^2^ = −0.825, *P* = 0.022) is interesting but is still not conclusive, leaving the adaptive significance of the response open. Therefore, the mechanisms typically associated with the regulation of aerobic metabolism in crustaceans^6,7^ during exposure to acutely declining PO_2_ cannot account for the oxyregulatory capacity observed in individuals exposed to hypoxia for one week. Therefore we explored morphological and physiological changes typically associated with an ability to regulate aerobic metabolism during prolonged hypoxic exposure in crustaceans^6,7,21,22^. There was no evidence of gill remodelling in *G. chevreuxi* after one week of hypoxic exposure. Such remodelling has been shown to be a mechanism used to maximise O_2_ uptake in some aquatic animals in response to prolonged exposure to hypoxia^23,24^. Population differences in gill area are documented in the related *Gammarus duebeni*^25^ and *Gammarus minus*^26^. However, the extent to which these population differences are driven by phenotypically plastic responses, particularly with regards to exposure to PO_2_, has not been explored.

Although we found no evidence of hypoxia-induced changes in respiratory pigment concentration in *G. chevreuxi*, at the transcription level, GO term analysis indicated a significant upregulation of genes enriched for “oxygen transporter activity” including genes putatively identified as haemocyanin. The seeming mismatch between haemocyanin transcript and protein expression levels has been previously documented in crustaceans upon exposure to hypoxia, and attributed to slow protein turnover^27^. In fact, it was recently shown that hypoxia can decrease fractional protein synthesis rates in the shrimp *Litopenaeus vannamei* through a reduction in RNA translational efficiency, and not RNA capacity^28^. Altering mechanisms of O_2_ transport has been previously described as an effective mechanism in maintaining O_2_ uptake at the respiratory surfaces and O_2_ delivery at the tissues during hypoxic exposure even in small animals such as amphipods^29^. The oxygen transport mechanisms by which oxyregulation can be achieved operate on two different timescales. Hypoxia can acutely modulate haemocyanin O_2_ affinity *via* allosteric effectors such as *L*-lactate or, upon prolonged exposure, modify extrinsic pigment O_2_ affinity and/or total haemocyanin concentration^6,30^. Crustaceans synthesize a range of haemocyanin subunits, assembled into functional oligomers with different intrinsic O_2_ affinities. The hypoxia-related shifts in expression of haemocyanin genes observed in this study, suggest differential isoform expression, possibly leading to the synthesis of different subunits under normoxia and hypoxia, indicating altered O_2_ affinity through structural changes upon exposure to hypoxia^30–33^. As we currently have no information on short-term modulation this option must remain open. In conclusion, *G. chevreuxi* appear to cope with hypoxia by employing mechanisms typically associated with prolonged exposure after just one week.

F_0_ adults exposed to moderate hypoxia for one week appear to be acclimated to the experimental conditions, as suggested by the maintenance of metabolic performance under moderate hypoxia, as well as the maintenance of both rates of O_2_ uptake and P_c_ under declining PO_2_. This is consistent with previous work on gammarids, where metabolic acclimation, defined as the reduction in rates of O_2_ uptake following an initial increase in response to environmental stress, occurs within 2–3 days (reviewed by^34^). However, the short-term acclimation ability observed in adults after one week does not predict acclimation potential in adults of *G. chevreuxi* reared under moderate hypoxia from hypoxia-exposed parents. Irrespective of treatment, F_1_ juveniles displayed significantly greater rates of mass-specific O_2_ uptake and poorer oxyregulation (i.e. greater P_c_ values) when exposed to acutely declining PO_2_ than their parents. The ontogeny of the ability of crustaceans to maintain respiratory independence under such conditions is poorly studied compared with our knowledge of the pattern and mechanisms underpinning such regulation in adults. Many adult crustaceans, including amphipods^13^, display a marked ability to oxyregulate down to comparatively low PO_2_. In most cases, this ability is not present throughout the individual’s life cycle, but develops during ontogeny^35–38^. It is difficult to know whether the higher P_c_ observed in *G. chevreuxi* juveniles, both under normoxia and hypoxia, is attributable to qualitative developmental changes rather than concomitant quantitative changes in body size, which are also known to affect the P_c_ value^12,39^. Despite this poorer oxyregulatory ability, juveniles reared under hypoxia from hypoxia-exposed parents showed evidence of acclimation to low PO_2_, as they maintained similar metabolic performance under declining PO_2_ as juveniles reared under normoxia. Under normoxic conditions, when F_1_ juveniles bred from normoxia-exposed parents reached the adult stage, O_2_ uptake and P_c_ were similar to levels characteristic of F_0_ amphipods. In contrast, F_1_ adults bred from hypoxia-exposed F_0_ parents and reared under hypoxia did not acclimate and had higher O_2_ uptake and P_c_ levels than F_0_ amphipods. Higher P_c_ levels in adults reared under hypoxia compared to normoxia indicate loss of respiratory independence from the environment at higher oxygen levels, thus poorer oxyregulatory ability. This suggests that adults reared under hypoxia do not develop the adult pattern of respiratory regulatory capacity. In fact, under hypoxia, P_c_ in F_1_ adults increased to a value greater than that of the F_1_ juveniles, indicating that the adult has lost the moderate oxyregulatory capacity it possessed as a juvenile. This suggests a detrimental effect of hypoxia in individuals reared under low PO_2_, not apparent upon exposure of adults.

Exposure to moderate hypoxia did not result in reduction in fitness of *G. chevreuxi* when expressed in terms of number of offspring produced and developmental time. While there was no effect of hypoxia on maternal investment in terms of egg volume, offspring of hypoxia-exposed parents were significantly smaller than those of normoxic parents. Maternal state can influence the energy allocation to the offspring, thereby altering survival, growth rates and fecundity^40^. The ability to alter brood size and egg composition has been implicated in acclimation responses to hypoxia, by influencing the offspring phenotype^41^. It is possible that the observed reduction in body size under hypoxia is a result of such alterations. While, in some species, large adults appear to be significantly less tolerant to hypoxia than small individuals^42,43^, the relationship between size and hypoxia resistance remains controversial^41^. The hypoxia-induced differences in hatchling size observed in this study persisted into sexual maturity. Given the reduced regulatory ability in hypoxia-exposed adults, the observed hypoxia-related reduction in body size appears maladaptive. It is possible that maternal investment, in terms of egg composition, is also affected by hypoxia-induced starvation in females, leading to reduced quality of egg contents and concomitant effects on offspring growth.

We suggest that the increased prevalence of hypoxia in coastal regions will have marked effects on some aquatic invertebrates that would currently be classed as tolerant, based on their ability to strongly regulate aerobic metabolism upon short-term exposure to hypoxia. Consequently, it is likely that we will underestimate the number of vulnerable species in an affected region if we rely on data from short-term studies. This said, the potential for transgenerational acclimation upon chronic exposure of the parental generation and/or genetic adaptation through generations cannot be excluded as mechanisms of survival. Recent work has demonstrated that a range of negative effects of environmental change can be mitigated in offspring of parents exposed to the same environmental conditions through transgenerational acclimation^41,44–51^. While in the present study, the parental generation was exposed to hypoxia at the time at which the fertilization of their egg occurred, longer periods of parental exposure than those used in this study could alleviate the effects of hypoxia in the future generations in this species through transgenerational acclimation (see^52^). Some of these mechanisms are currently under investigation in our laboratory.

## Materials and Methods

### Amphipod collection, pre-exposure and treatment conditions

Amphipods were collected during low tide using a kick net (mesh size = 500 µm) from the Plym estuary, Devon (50° 23′ 24′′ N, 4° 5′ 7′′ W) in spring 2013 and 2014, returned to a temperature-controlled laboratory and sorted into stock aquaria containing diluted sea water (vol. = 7.5 L, T = 15 °C, S = 15, O_2_ = 100% air saturation (air saturation) light = 12 h:12 h L:D cycle). Amphipods were held in the stock aquaria in pre-exposure conditions for a minimum of two weeks, and fed carrot *ad libitum*. Carrots have been used previously as feed for amphipods and contain slightly greater concentrations of copper than the seaweeds consumed by these animals in nature; thus copper concentration in the food is unlikely to be a limiting factor in haemocyanin production. Aquaria were provided with a substrate of aquarium gravel to allow individuals to shelter and thereby minimise cannibalism. Water changes were performed weekly. Individuals were identified as *Gammarus chevreuxi* and sorted by sex at the end of the pre-exposure period according to^18^.

After this pre-exposure period, amphipods were kept under either normoxia (PO_2_ = 100% air saturation or 6.5 mL O_2_ L^−1^ at temperature = 15 °C, salinity = 15) or moderate hypoxia (PO_2_ = 40% air saturation or 2.6 mL O_2_ L^−1^ at temperature = 15 °C, salinity = 15) for one week (i.e. short-term exposure) as follows: 15 wild sexually matured (F_0_) pre-copula pairs were allocated to each of eight aquaria (vol. = 0.4 L) per treatment (normoxia = 100% air saturation or hypoxia = 40% air saturation) containing diluted sea water (T = 15 °C, S = 15, PO_2_ = 100 or 40% air saturation). Forty percent air saturation was chosen for the hypoxic treatment, to reflect the low end of the range experienced seasonally in local estuaries^53^, and also aligns with guideline levels for the use of the term ‘moderate hypoxia’^4^. Normoxia in control aquaria was maintained by bubbling ‘scrubbed’ air (air that had been previously been bubbled through 2 mol.L^−1^ NaOH) directly into the water through an airline, with the flow controlled by a flowmeter set to approximately 3.5 L min^−1^. Hypoxic conditions were maintained by bubbling a mixture of ‘scrubbed’ air and nitrogen, stabilised using flow valves set to 0.5 L min^−1^ and 0.75 L min^−1^ for air and nitrogen respectively. There were no significant differences in pH or carbon dioxide content (8.13 ± 0.11 and 2.47 ± 0.38 mmol L^−1^ respectively) between control and experimental treatments. After seven days, different groups of *G. chevreuxi* males of approximately equal size and not in amplexus were randomly selected from each treatment group and used in respirometry trials (n = 8 per treatment), measurement of gill area (n = 20 per treatment), analysis of haemolymph (n = 40 per treatment) and transcriptome profiling (n = 30 per treatment). In all cases, amphipods were selected from across the eight aquaria per treatment, thus minimising pseudoreplication. An overview of the measurements performed is given in Table S3.

### Respirometry, ventilation and perfusion upon short-term exposure to hypoxia under their respective O_2_ conditions and under declining PO_2_

Rates of O_2_ uptake of individuals were measured under their respective exposure conditions using closed, gas-tight, blackened-out, glass incubation chambers (vol. = 23 mL). Each chamber was supplied with filtered (22 μm), autoclaved diluted sea water and a magnetic flea. Individual males were restrained in a mesh envelope (2 mm mesh size), which separated the animal from the flea and ensured the animals naturally remained quiescent during the experiment, while allowing sufficient water flow through the mesh. Individuals were allowed to settle for 80 min in their unsealed chambers, at which time the PO_2_ of a water sample taken from within the chamber was measured using a microcell, containing an O_2_ electrode coupled to an O_2_ meter. Immediately after, chambers were sealed while submerged and placed onto a multi-channel magnetic stirrer to ensure mixing of water and to prevent stratification of O_2_ within the respirometer. A second sample was taken after 4.5 h (average period taken for the PO_2_ in the chamber to be reduced to approximately 80% of the original). Immediately before the water sample was extracted for O_2_ analysis, heart rate (HR) and pleopod beat frequency (PR) of control and hypoxia-exposed individuals were quantified twice over 15 s for each individual, manually under low magnification (<10×) light microscopy. Heart rate was measured by counting the number of contractions of the heart, visible through the carapace as a tubular structure running dorsally along the body. A pleopod beat was defined by a complete swing from the back of the pleon to the pereon parallel to the body. As the experimental exposure was standarised by time, there was some variability in PO_2_ at which these traits were measured (PO_2_ declined by 18.01 ± 3.25% in 4.5 h), but there were no significant differences in the amount by which PO_2_ declined between treatments (*P* = 0.521). The experiment was terminated by removing individuals from the chamber, gently blotting them dry, and weighing them. The difference between water PO_2_ within the chamber at the beginning and at the end of the experiment was used to calculate rate of O_2_ uptake, expressed as μL O_2_ mg wet mass^−1^ h^−1^ STP, and this was used as a proxy for resting metabolic rate (RMR).

Rates of O_2_ uptake of individuals under acutely declining PO_2_ were measured for individual F_0_ males after 7 d exposure to either normoxia or hypoxia. Measurements were carried out under the same conditions as before with the following modifications 1) for both treatments, water was aerated until air saturation was 100%, to expose individuals to declining PO_2_ starting at a common and comparable starting point (i.e. normoxia), and 2) once sealed, chamber water PO_2_ was measured every 15 min using a calibrated optical O_2_ sensor until O_2_ saturation reached 1–0%, to allow for estimation of P_c_ under declining PO_2_. Preliminary experiments established that there was no significant difference between mean rates of O_2_ uptake measured by the two different methods (i.e. polarographic electrodes and O_2_ optodes) when tested under the same conditions. Therefore, any differences in rates of O_2_ uptake between experiments can be attributed to differences resulting from the different conditions experienced by the animal during respirometry (i.e. a small reduction in PO_2_ versus acutely declining PO_2_)

### Estimation of haemocyanin to protein ratios

To estimate haemocyanin to protein ratios in the haemolymph, four pools of haemolymph (0.5 µL extracted, n = 5 individuals) were collected from individuals in each experimental treatment. This was done by inserting the needle of a microsyringe (vol. = 10 µL) directly into the heart, dorsally through the second and third dorsal plates of the pereon. Each pooled sample was diluted with deionised water (1:20) and absorbance was measured at λ = 278 nm (protein peak) and 336 nm (copper peak) using a UV-VIS Spectrophotometer. Absorbance (Abs) ratios (336/278) were used as proxies for haemocyanin to protein ratios.

### Measurement of gill surface area

Measurements of total gill surface area were performed for a wide size range of individuals (n = 20 per treatment) per treatment exposed to either normoxia or hypoxia^54^. The area of an individual gill was calculated as twice its planar projection. Body length of amphipods from the rostrum tip to the telson posterior margin was measured using the same method, and converted to dry mass by interpolation from a regression equation (y = a + bx, where y = dry mass (mg) and x = length (mm)) fitted to the length/dry mass relationship (*R*^2^ = 0.773, df = 19, *P* < 0.001).

### Transcriptome profiling

Transcriptome profiling using Next Generation Sequencing (NGS) was performed for individuals exposed to normoxia and hypoxia for one week. Three replicate pools of males (n = 10) for each treatment were fast frozen in liquid nitrogen, and immediately stored at −80 °C. Total RNA was isolated using a PureLink RNA Mini Kit (ThermoFisher Scientific, UK). TruSeq RNA libraries (Illumina, USA) were synthesised and sequenced using 100 base paired-end sequencing (HiSeq 2000, Illumina, USA). Details of assembly, annotation and data availability are provided in^55^. Alignment of the assembled transcriptome to the original reads was performed using Bowtie v.1.1.1^56^. Gene counts were generated by RSEM v.1.2.29 and imported into R v.3.3.1 using Tximport v.1.0.3. Differential gene expression analysis was performed at the level of Trinity “genes” using DESeq2.v.1.12.4^57^ to identify haemocyanin genes that displayed significantly different expression (*P* < 0.05) between the hypoxic and normoxic treatment.

### Estimation of fitness under different O_2_ regimes

Breeding stocks were maintained in both normoxic and hypoxic conditions and replenished monthly with freshly-caught amphipods over a four-month period (12 h:12 h L:D cycle, temperature = 15 °C, salinity = 15, PO_2_ = 100 or 40% air saturation). Pre-copula pairs were exposed to treatment conditions until the pair separated and for a minimum of one week. At this point, the male was removed, and females carrying eggs were isolated. For assessment of brood size and egg volume, a subset of isolated females was used from both treatments (42 broods, 236 eggs) for examination within 24 h of fertilization. Eggs were carefully removed from the brood pouch and the length and width of each egg was measured using ImageJ and used to calculate egg volume as an oblate ellipsoid. To estimate developmental time and size at hatching, a second sub-set of the isolated females were monitored daily until the eggs hatched, at which point the total time from fertilization was recorded. Hatchlings were counted and imaged, and their body length was measured as previously described.

### Respirometry upon chronic exposure to hypoxia across the life-cycle under declining oxygen tensions

The remaining F_0_ amphipods exposed to normoxia and hypoxia for at least a week, which were not used to make the measurements described above, remained under either normoxia or moderate hypoxia and were allowed to mate. Their offspring (F_1_) were reared under the same conditions as their parents until they reached either a juvenile stage (F_1_ juveniles, six weeks post-hatching) or sexual maturity (F_1_ adults), defined by the onset of egg production (i.e. chronic exposure across their life cycle). Rates of O_2_ uptake of individuals under acutely declining PO_2_ were measured for F_1_ juveniles and F_1_ male adults reared under either normoxia or hypoxia. Measurements were carried out under the same conditions as before, but the volume of the chambers was adjusted to 15 mL and 1.28 mL for adults and juveniles respectively to adjust for body size at different life cycle stages. An overview of the measurements performed is given in Table S3.

### Statistical analyses

All statistical analyses were carried out using R v.2.3. For all analyses, data were tested for normality and/or variance homogeneity. Appropriate transformations were applied as required. Statistical significance was assigned at *P* ≥0.05. Data are expressed as mean ± s.e.m.

Three t-tests were conducted to compare the effect of oxygen regime (100 and 40% air saturation) on 1) mean mass-specific rates of O_2_ uptake, 2) mean heart rate, and 3) mean ventilation rate. Two two-way ANOVA were conducted to compare the main effects of O_2_ regime (100 and 40% air saturation), generation group (F_0_, F_1_ juveniles and F_1_ adults) and their interaction on 1) mean mass-specific oxygen uptake, and 2) P_c_. Subsequent Tukey’s *post hoc* analyses were used to detect significant differences between groups. The effect of treatment on mean haemocyanin to protein ratios was tested using Student’s t-test. Gill area and lamellar area were compared using ANCOVA, with amphipod dry mass as the covariate. To determine the effect of the PO_2_ on brood size, developmental time, egg volume and time at hatching, pairwise treatment comparisons were performed using either t-tests or Mann-Whitney U tests.

## Electronic supplementary material

Supplementary information
