# Supplementary material for: Short-term acclimation in adults does not predict offspring acclimation potential to hypoxia
Source: Sci Rep. 2018 Feb 16;8:3174. doi: 10.1038/s41598-018-21490-y (PMC5816618; doi:10.1038/s41598-018-21490-y)
Supplement: Supplementary file 1 — Supplementary information [file 41598_2018_21490_MOESM1_ESM.pdf]

Supplementary information for: Short-term acclimation in adults does not predict offspring acclimation potential to hypoxia

Manuela Truebano*, Oliver Tills, Michael Collins, Charlotte Clarke, Emma Shipsides, Charlotte Wheatley, John I. Spicer

Table S1. Annotation and expression data of ten differentially expressed genes putatively identified as haemocyanin using the most significant match (lowest E-value) generated by BLAST searches (BlastP and BlastX) against the UniProt/Swissprot database. Gene expression data for each of the hemocyanin genes are presented as TMM-normalised transcripts per million (TPM). Log-2 fold changes (FC) and associated probability (adjusted P-value, P_{adj}) are also shown.

**Contig**	**Annotation**	**Accession number**	**Identity (%)**	**E-Value**	**Gene expression (TPM)**						**Log_2 FC**	**P_{adj}**
					**100%_1**	**100%_2**	**100%_3**	**40%_1**	**40%_2**	**40%_3**		
DN65240_c0_g2	HCY_PALVU	P80888	62.25	0	0.38	0.14	0.03	0.51	5.40	2.974	3.91	1.34e-04
DN68344_c0_g5	HCY_PALVU	P80888	76.98	3e-165	2.26	2.30	1.52	5.64	50.33	31.29	3.89	7.85e-08
DN69931_c0_g7	HCY_PALVU	P80888	55.48	2e-118	6.71	5.86	2.77	21.01	180.21	114.21	4.38	1.27e-09
DN69931_c0_g8	HCY_PALVU	P80888	52.68	5e-76	4.43	3.61	1.51	15.79	108.97	65.65	4.36	1.19e-09
DN70919_c0_g2	HCYA_APHSP	P14750	44.75	4e-88	11.65	9.11	3.33	2.74	2.25	1.54	-1.59	2.30e-02
DN65240_c0_g1	HCYA_PANIN	P04254	62.46	0	0.12	0.44	0.00	1.18	4.81	2.88	3.94	8.35e-05
DN69931_c0_g3	HCYA_PANIN	P04254	58.70	4e-13	20.22	26.08	14.54	77.74	468.65	296.78	3.88	1.38e-09
DN69931_c0_g4	HCYB_ASTLP	P83180	62.50	2e-09	7.50	3.20	7.19	22.26	130.92	86.11	3.81	9.41e-07
DN69931_c0_g6	HCYB_ASTLP	P83180	57.50	3e-08	0.00	0.30	0.00	0.15	7.60	4.42	4.79	7.23e-03
DN68344_c0_g1	HCYB_PANIN	P10787	60.67	3e-30	0.87	3.83	2.24	8.24	20.17	12.21	2.77	4.29e-03

Table S2. Results of two-way ANOVA for the effects of life cycle stage, O_2 regime (normoxia-100 % air saturation, hypoxia- 40 % air saturation) and their interaction on mass specific rates of O_2 uptake and critical O_2 tension (P_c).

Response	Factor	df	Sum Sq	Mean Sq	F-value	P-value
O_2 uptake	Life cycle stage	2	13.336	6.668	41.67	<0.001
	O_2 regime	1	0.136	0.136	0.85	0.365
	Stage x O_2	2	1.364	0.682	4.26	0.026
	Residuals	25	4.000	0.160		
Pc	Life cycle stage	2	35.13	17.56	23.58	<0.001
	O_2 regime	1	8.83	8.833	11.86	0.002
	Stage x O_2	2	23.49	11.745	15.77	<0.001
	Residuals	25	18.62	0.745		

Table S3. Overview of the different variables measured in each generation and life-cycle stage. All measurements were taken in individuals exposed to normoxia (100 % air saturation) or hypoxia (40 % air saturation)

Generation	Life- cycle stage	Exposure time	Measurements taken
F_0	Adult	One week	RMR<br>Heart rate<br>Ventilation rate<br>Gill surface area<br>Haemocyanin:protein<br>Haemocyanin isoform expression
F_0 to F_1		> One week	Fitness (brood size, egg volume, developmental time and size at hatching of offspring)
F_1	Juvenile	Embryonic development and six weeks post-hatching.	O_2 uptake under declining PO_2<br>Pc
F_1	Adult	Until individuals reached sexual maturity (approx.3 months)	O_2 uptake under declining PO_2<br>Pc
